# Networking of Public Health Microbiology Laboratories Bolsters Europe’s Defenses against Infectious Diseases

**DOI:** 10.3389/fpubh.2018.00046

**Published:** 2018-02-26

**Authors:** Barbara Albiger, Joana Revez, Katrin Claire Leitmeyer, Marc J. Struelens

**Affiliations:** ^1^Scientific Advice Coordination Section, Office of the Chief Scientist, European Centre for Disease Prevention and Control, Stockholm, Sweden; ^2^Microbiology Coordination Section, Office of the Chief Scientist, European Centre for Disease Prevention and Control, Stockholm, Sweden

**Keywords:** public health laboratory networks, public health microbiology, external quality assessment, molecular diagnostics, antimicrobial susceptibility testing, molecular typing, communicable disease surveillance, emerging diseases

## Abstract

In an era of global health threats caused by epidemics of infectious diseases and rising multidrug resistance, microbiology laboratories provide essential scientific evidence for risk assessment, prevention, and control. Microbiology has been at the core of European infectious disease surveillance networks for decades. Since 2010, these networks have been coordinated by the European Centre for Disease Prevention and Control (ECDC). Activities delivered in these networks include harmonization of laboratory diagnostic, antimicrobial susceptibility and molecular typing methods, multicentre method validation, technical capacity mapping, training of laboratory staff, and continuing quality assessment of laboratory testing. Cooperation among the European laboratory networks in the past 7 years has proved successful in strengthening epidemic preparedness by enabling adaptive capabilities for rapid detection of emerging pathogens across Europe. In partnership with food safety authorities, international public health agencies and learned societies, ECDC-supported laboratory networks have also progressed harmonization of routinely used antimicrobial susceptibility and molecular typing methods, thereby significantly advancing the quality, comparability and precision of microbiological information gathered by ECDC for surveillance for zoonotic diseases and multidrug-resistant pathogens in Europe. ECDC continues to act as a catalyst for sustaining continuous practice improvements and strengthening wider access to laboratory capacity across the European Union. Key priorities include optimization and broader use of rapid diagnostics, further integration of whole-genome sequencing in surveillance and electronic linkage of laboratory and public health systems. This article highlights some of the network contributions to public health in Europe and the role that ECDC plays managing these networks.

## Introduction

Facing global epidemics of infectious diseases and rising multidrug resistance, microbiology laboratories provide pivotal information trough surveillance, from local to global levels, as specified in the International Health Regulations (1). At international level, the World Health Organization (WHO) operates laboratory networks that are part of epidemic preparedness and response programs as well as monitor communicable disease elimination and eradication programs (2, 3). In the European Union (EU), the European Centre for Disease Prevention and Control (ECDC), a public health agency financed by the EU, is tasked with detection, surveillance, and risk assessment of threats to human health from communicable diseases (4, 5). ECDC has a multidisciplinary workforce providing scientific advice, epidemic intelligence, disease surveillance, outbreak response support, preparedness support, microbiology support health communication, and training activities in collaboration with public health experts and national agencies in EU countries. It does not operate its own microbiology laboratories but relies instead on laboratory information provided at national level. EU countries report notifiable diseases to ECDC using EU case definitions (6). ECDC is mandated to “foster the development of sufficient capacity within the Community for the diagnosis, detection, identification, and characterization of infectious agents which may threaten public health, by encouraging cooperation between expert and reference laboratories” (4). This mandate builds upon decades of professional collaboration in Europe between infectious disease experts, microbiologists, and epidemiologists.

This article highlights ECDC key activities supporting the coordination of laboratory networks targeting the diseases which ECDC monitors at EU level. It discusses the effectiveness of laboratory response across Europe to recent public health events and indicates future directions for enhancing public health microbiology.

## ECDC’s Support to EU Member States Laboratory Capacities

Since 2007–2010, ECDC has gradually supported the coordination of 12 EU-wide networks of microbiology laboratories embedded in disease specific networks. These primarily contribute to integrated epidemiological and microbiological surveillance for EU notifiable communicable diseases as well as to detection of emerging diseases (Table 1). Within these networks, ECDC supports microbiology activities ranging from EU-wide laboratory network coordination, capability and external quality assessments (EQA), laboratory staff training, reference microbial strain collections establishment, supranational reference services, outbreak investigations and risk assessments support, technology assessment, method harmonization and development of standard procedures, and integration of molecular typing into surveillance programs. Since 2014, ECDC also operated the EU laboratory capability (EULabCap) system for monitoring the capacities and capabilities of microbiology laboratories in EU countries (7).

**Table 1:** European Union (EU) public health microbiology laboratory networks supported by ECDC by disease group or health issue, 2017.

Antimicrobial resistance and healthcare-associated infections
**European Antimicrobial Resistance Surveillance Network (EARS-Net):** The EARS-Net is a network of national surveillance systems providing reference data on antimicrobial resistance in invasive bacterial pathogens from clinical laboratories in the EU/EEA

**Healthcare-associated Infections Surveillance Network—Supporting capacity building for the surveillance of *Clostridium difficile* infections (HAI-Net CDI):** Outsourced microbiological support to hospital-based surveillance of CDI aims to increase the capacity of laboratories in EU/EEA Member States to (1) perform CDI diagnostic practices with high diagnostic accuracy and (2) acquire comparable typing data from *C. difficile* isolates

**Emerging and vector-borne diseases**

**Emerging Viral Diseases-Expert Laboratory Network (EVD-LabNet):** The EVD-LabNet is strengthening capacity for early detection and surveillance of (re)emerging viral diseases in the EU/EEA countries and EU Candidate Countries. It provides scientific advice to ECDC and works in close collaboration with other Commission initiatives. Formerly named “European Network for Diagnostics of Imported Viral Diseases” (ENIVD)

**Food- and waterborne diseases, zoonoses**

**European Food- and Waterborne Diseases and Zoonoses Network (FWD-Net):** The FWD-Net network advises ECDC and contributes to strengthening surveillance and prevention of 21 food- and waterborne diseases and zoonoses in the EU/EEA, in close collaboration with EFSA, WHO and global public health partners. Activities include microbiology capacity building, EQA schemes, and harmonization of laboratory-based surveillance including molecular/genomic typing

**European Legionnaires’ Disease Surveillance Network (ELDSNet):** The ELDSNet carries out surveillance of Legionnaires’ disease in the EU/EEA and supports microbiology capacity building, including diagnostics and molecular typing, in close collaboration with WHO and global public health partners

**Creutzfeldt–Jakob Disease International Surveillance Network (EuroCJD):** The EuroCJD is coordinated from the National CJD Surveillance Unit in Edinburgh with funding by the ECDC. It provides advanced diagnostic services for those Member States that lack diagnostic capability for transmissible encephalopathies and carries out surveillance of variant Creutzfeldt**–**Jakob disease (vCJD) in the EU/EEA

**HIV, sexually transmitted infections and viral hepatitis**

**European Gonococcal Antimicrobial Surveillance Programme (Euro-GASP):** The Euro-GASP network carries out sentinel surveillance of gonococcal antimicrobial resistance in the EU/EEA and is strengthening capacity for gonococcal culture and antimicrobial susceptibility testing through laboratory training and EQA schemes. In addition the network performs molecular typing of *Neisseria gonorrhoeae*

**Influenza**

**European Reference Laboratory Network for Human Influenza (ERLI-Net):** The ERLI-Net sub-network of reference laboratories of the European Influenza Surveillance Network carries out virological surveillance of human influenza in the EU/EEA and strengthening laboratory capacity for influenza virus detection, antiviral susceptibility testing, and typing, in close collaboration with WHO

**Tuberculosis**

**European Reference Laboratory Network for Tuberculosis (ERLTB-Net):** The ERLTB-Net sub-network of reference laboratories from EU/EEA Member States supports harmonization of methods and laboratory capacity for tuberculosis diagnosis, antimicrobial susceptibility testing and molecular/genomic typing

**Vaccine preventable diseases**

**European Pertussis Laboratory Network (EUPert-LabNet):** The EUPert-LabNet aims to improve harmonization of methods and support laboratory capacity for pertussis diagnosis as well as support the integration of epidemiological and laboratory surveillance in the EU/EEA

**Invasive Bacterial Disease Laboratory Network (IBD-LabNet):** The IBD-LabNet network integrates epidemiological and laboratory surveillance for invasive bacterial infections caused by *N. meningitidis, H. influenzae*, and *S. pneumoniae*. The network focuses on strengthening reference laboratory capacity in the EU/EEA, to support the integration of epidemiological and laboratory surveillance

**Diphtheria Laboratory Network (Diphtheria-LabNet):** The Diphtheria-LabNet aims to assess and improve laboratory performance through standardized and appropriate methods for laboratory diagnosis of diphtheria as to ensure accurate and comparative diphtheria surveillance across Europe. The network also aims to expand knowledge on serological immunity procedures for detecting diphtheria antitoxin antibodies

To strengthen the quality of surveillance and threat detection, ECDC has commissioned in the last 7 years 121 EQA exercises for it’s laboratory network members (Table 2). These EQAs covered a range of methods from pathogen detection and/or identification, molecular typing, to antimicrobial susceptibility testing (AST). EQAs was rated as one of the highest valued ECDC capacity building activities by external stakeholders (8), with its certificates being used for laboratory accreditation at the national level. EQA results over the years indicate better performance across countries, even though gaps remain (9–12). Having the networks centralized at ECDC has facilitated harmonization of EQA practice and cost efficiency across networks.

**Table 2:** Overview of ECDC- supported laboratory external quality assessments schemes by Disease Programme (DP), target pathogen, testing area and year, 2010–2016.

DP^a^	Pathogen(s)	Testing area for assessment^c^					Number of EQA exercises/year^d^						
		Detection	Typing	Virulence/resistance	AST	Reporting	2010	2011	2012	2013	2014	2015	2016
ARHAI	*Enterococcus faecalis, E. faecium, E. coli, K. pneumoniae, P. aeruginosa, S. pneumoniae, S. aureus, Acinetobacter* spp.^b^	×			×	×	1	1	1	1	1	1	1
	Methicillin-Resistant *Staphylococcus aureus*		×			×	1						
	*Clostridium difficile*		×			×		1		1	1		
	Carbapenemase-producing *Enterobacteriaceae*			×		×				1			

EVD	Hantavirus	×					1						
	Yellow fever virus^e^	×					2						
	Crimean Congo Hemorrhagic Fever Virus	×						1					
	Tick-borne encephalitis virus	×						1					
	West Nile fever virus^e^	×						2	1				
	Rift valley fever virus	×							1				
	Dengue virus	×								1			
	Lassa virus	×								1			
	Middle East respiratory syndrome coronavirus	×									1		
	Chikungunya virus^e^	×									2		
	Zika virus	×											1

IRV	Human influenza virus^f^	×			×	×	2			2		2	

FWD	*Salmonella enterica*		×		×		2		1	1	2	1	1
	Shiga toxin-producing *E. coli*		×	×			2		1		2	1	
	*Listeria monocytogenes*		×							2	1	1	1
	*Campylobacter jejuni* and *C. coli*				×						1		1
	variant Creutzfeldt–Jakob	×				×	1		1	1			1
	*Legionella pneumophila*	×	×				4	4	4	4		4	

HSH	*Neisseria gonorrhoeae*				×	×	1	1	1	1	1	1	1

VPD	*Corynebacterium diphtheriae*	×	×				1		2	1			
	*Neisseria meningitidis*	×	×		×			1	1		1		1
	*Haemophilus influenzae*	×	×	×				1	1		1		1
	*Streptococcus pneumoniae*	×	×		×		1		1		1		1
	*Bordetella pertussis*	×	×				1		2	1			1

TB	*Mycobacterium tuberculosis*^g^	×	×		×	×	2	2	1	2	2	2	2

Total							22	15	19	20	17	13	13

*^a^DP, ECDC Disease Programme; ARHAI, Antimicrobial Resistance and Healthcare-Associated Infections Programme; EVD, Emerging and Vector-borne Diseases Programme; IRV, Influenza and other Respiratory Viruses Programme; FWD, Food and Waterborne and zoonoses Diseases Programme; HSH, HIV, Sexually Transmitted Infections and viral Hepatitis Programme; VPD, Vaccine Preventable Diseases Programme; TB: Tuberculosis Programme*.

*^b^EQA cover at least six pathogens included by EARS-Net and resistance phenotypes relevant to the current epidemiology in the EU/EEA*.

*^c^*Detection*: diagnostic test (using culture, molecular, or serological methods) and/or pathogen species identification; *Typing*: serotyping, molecular, or WGS typing; *Virulence/resistance*: identification of genetic/phenotypic determinants; *AST*, Antimicrobial susceptibility testing; *Reporting*: data analysis and interpretation for reporting according to international/EU case definitions and standard nomenclature*.

*^d^For each pathogen, the number of exercises is dependent on the panel of strains/samples distributed*.

*^e^For these agents, one EQA focused on detection by PCR and another on serology*.

*^f^For influenza virus, one EQA focused on detection by PCR and virus culture with antigenic and genetic characterization and another EQA focused on antiviral susceptibility testing. In addition, WHO provided yearly EQA on PCR detection*.

*^g^For Mycobacterium tuberculosis, one EQA focused on conventional diagnostic tests and another on molecular typing*.

## Rapid Detection of Emerging Infectious Threats, 2012–2017

At the request of the European Commission or the Member States, EU laboratory networks together with ECDC participate in investigations and risk assessment of potential cross-border health threats caused by emerging diseases or outbreaks. Recent events illustrate how laboratory detection capacity developed across networks contributed to a coordinated public health management of threats in Europe and beyond.

In September 2012, a novel coronavirus was isolated from two cases of acute severe respiratory illness who had traveled to or resided in Saudi Arabia (13, 14). Within a few weeks, more cases were identified in patients with links to the Middle East. This virus was named Middle East respiratory syndrome coronavirus (MERS-CoV) (15). In November 2012, real-time reverse-transcriptase (RT)-polymerase chain reaction (PCR) detection and identification tests were developed to ensure rapid detection capability (14). Technical protocols and positive RNA control material were made available by the European Commission funded European Virus Archive project and distributed within the former ECDC funded “European Network for Diagnostics of Imported Viral Diseases” (ENIVD). ECDC, in collaboration with WHO Regional Office for Europe (WHO/Europe), surveyed detection capability for MERS-CoV by virology reference laboratories (16). Ten months after the virus discovery, laboratories had diagnostic capabilities in 24 of the 30 EU and European Economic Area (EEA) countries (17). In 2013, an ECDC EQA exercise showed correct performance in laboratories across the ENIVD (18). In 2015, 28 EU/EEA countries had capability to screen and confirm MERS-CoV cases for appropriate management (7). By October 2015, 14 cases of MERS had been diagnosed across seven EU countries among patients with connection with the Middle-East. Thanks to rapid diagnosis, patients were promptly isolated and secondary transmission to household members or hospital patients occurred only rarely (19).

In March 2013, fatal cases of human infection with novel reassortant avian influenza virus strain A(H7N9) following contact with infected poultry were reported in China (20). This was the first time that human infection and deaths due to a low-pathogenicity avian influenza virus had been identified. Within 1 month of this event, ECDC, the European Reference Laboratory Network for Human Influenza (ERLI-Net) coordinated by ECDC, and the WHO/Europe released a joint technical note on diagnostic preparedness for detection of these viruses (21). In May 2013, the capability of ERLI-Net laboratories to detect and subtype the novel avian influenza A(H7N9) viruses was jointly assessed by ERLI-Net, ECDC, and WHO/Europe (22). The survey showed that the generic influenza A virus detection and H7 and N9 subtyping assays used in 24 laboratories in 19 EU/EEA countries were adequate. Later in 2013, the results of an ECDC EQA confirmed that 33 of the 36 ERLI-Net laboratories correctly detected, typed, and subtyped the novel A(H7N9) influenza viruses (23). In 2015, EULabCap survey results documented that diagnostic capability for avian influenza A(H7N9) virus infection existed in 28 of 29 EU/EEA countries, suggesting that the ERLI-Net response support had facilitated European-wide laboratory compliance with ECDC/WHO influenza surveillance guidance (7). No human influenza case caused by this strain has yet been diagnosed in Europe but annual epidemics of human infections in China indicate a persisting risk.

In December 2013, the largest ever epidemic of Ebola virus disease (EVD), started in Guinea and quickly spread to neighboring West African countries where it caused over 10,000 deaths until controlled March 2016. In March 2014, as part of global assistance efforts, the EU allocated funding and deployed medical and laboratory staff and supplies in West Africa. The European Mobile field Laboratory (EMLab), an initiative by the International Cooperation and Development Office of the European Commission, established field diagnostic facilities in the affected countries to support patient screening (24). Between March 2014 and October 2015, more than 19,000 samples were tested in these laboratories (24). Deployment of an EMLab unit close to an Ebola Treatment Unit decreased the average turnaround time from reception of a sample to diagnostic result to 4 h instead of several days. Several EU-funded laboratory networks worked to ensure capacity for testing suspected cases of EVD both in Africa and in travelers returning from the epidemic affected areas. These included the ENIVD network and the Joint Action “Quality Assurance Exercises and Networking on the Detection of Highly Infectious Pathogens” (QUANDHIP) which was co-funded by the European Commission and Member States. Both networks contributed expert staff to the EMLab activities in Africa. The ENIVD members participated in an ECDC EQA exercise for rapid molecular diagnosis of EVD (25). Until September 2015, QUANDHIP expert laboratories provided tests for 692 patients with suspected EVD in Europe (26). During the epidemic, five cases of EVD were confirmed in repatriated patients from West Africa and three new cases were diagnosed in the EU, including two travel-associated cases and one nosocomial case in a healthcare provider (27). These data confirmed that EVD patient isolation measures based on rapid diagnostics interrupt virus transmission and mitigate the risk of spread from patients evacuated to EU countries.

In 2015, a novel healthcare-associated disease was discovered in Switzerland by Sax et al., who reported a hospital outbreak of cardiovascular infection caused by *Mycobacterium chimaera* in surgery patients, linked to contaminated heater-cooler units used in surgery (28). This environmental mycobacterium is difficult to detect (i.e., fastidious) and identify (i.e., need for DNA sequence analysis). In April 2015, following the report of similar cases of infection in the Netherlands, Germany and the UK, ECDC evaluated the risk across Europe (29). It advised performing diagnostic investigations to ascertain possible cases of post-surgical infections by this organism (29). In collaboration with experts from the affected countries, ECDC followed-up national investigations and published an EU case-definition and technical protocol for case detection, laboratory diagnosis, environmental testing, and molecular typing of *M*. *chimaera* infection (30). This protocol provided a basis for harmonized data collection across Europe to facilitate the sharing of information on the extent and the molecular epidemiology of the outbreak. In 2015 and 2016, *M. chimaera* strains were included in the EQA panel of the ECDC coordinated European Reference Laboratory Network for Tuberculosis (ERLTB-Net). An updated risk assessment by ECDC indicated that, by November 2016, 52 cases of invasive cardiovascular infection by *M. chimaera* had been documented across seven European countries (31). Investigations using whole-genome sequencing (WGS) of clinical and environmental isolates were conducted in several countries by national public health agencies in collaboration with device manufacturers. Their concordant findings consistently pointed to airborne dispersal of *M. chimaera* from a particular model of heater–cooler device as the most likely infection source in most cases. The device was found to be contaminated at the manufacturing plant with *M. chimaera* of the same genotype as in outbreak-related patients (32–34). Following replacement or repositioning of the device in the operating theater, no further case has been detected in the affected surgical care facilities so far.

## Advancing Laboratory-Based Surveillance for Communicable Diseases and Antimicrobial Resistance

To overcome inconsistency in categorizing antimicrobial resistance across countries, the European Committee for Antimicrobial Susceptibility Testing (EUCAST) operating under the joint auspices of ECDC and the European Society of Clinical Microbiology and Infectious Diseases has developed standard methods and nomenclature and advocated their use for *in vitro* AST of bacteria and fungi of medical interest. They have published evidence-based criteria for categorization of clinical isolates as wild-type/non-wild phenotype or clinically susceptible/resistant to antimicrobial agents (35). In 2012, EU case definitions enforced the EUCAST clinical breakpoints for surveillance of antimicrobial resistance in humans (6). Between 2011 and 2015, use of EUCAST breakpoints has rapidly progressed in clinical microbiology laboratories across Europe (36). The percentage of EU clinical laboratories reporting to the ECDC coordinated European Antimicrobial Resistance Surveillance Network (EARS-Net) which are using EUCAST susceptibility breakpoints increased from 29 to 84% over this 5-year period (37, 38). Likewise, the percentage of national reference laboratories participating in the ECDC coordinated European Gonococcal Antimicrobial Surveillance Programme (Euro-GASP) that use EUCAST breakpoints increased from 62 to 85% from 2014 to 2016 (12, 39). This harmonization across laboratories, encouraged by ECDC, improves the quality of surveillance of antimicrobial resistance in Europe.

Because antimicrobial resistance in zoonotic pathogens is transferable from food animals to humans, One-Health monitoring, covering microbiota from human, animal, and environmental sectors in a holistic approach, is essential for containment. Unfortunately, comparisons between antimicrobial resistance data from humans, food, and animals have long been hampered by the use of different test methods and interpretative criteria in each health sector. Whereas AST results on human isolates is interpreted with clinical breakpoints, those from healthy animal and food isolates monitoring are interpreted based on epidemiological cut-off values (ECOFFs). These cut-off values distinguish normally susceptible isolates (wild-type phenotype) from those who have acquired resistance to the antimicrobial (non-wild-type phenotype). In 2013, ECDC together with national representatives from its Food- and Waterborne Diseases and Zoonoses (FWD) network developed an EU protocol for harmonized monitoring of antimicrobial resistance in *Salmonella enterica* and *Campylobacter jejuni* and *C. coli* from human isolates, aiming at increasing the quality and comparability of data collected in the EU Member States by ECDC with those collected by the European Food Safety Authority (EFSA) from the veterinary sector. This protocol defines the panel of antimicrobials, the test methods, and reporting of quantitative susceptibility data to allow direct comparison with animal isolates’ data interpreted with ECOFF values (40). Between 2013 and 2015, the proportion of reporting countries compliant with the EU protocol doubled from 30 to 60%, improving comparability of antimicrobial resistance surveillance data among sectors (41).

Limiting the dissemination of multidrug-resistant bacterial pathogens requires a better understanding of the emergence and mode of spread of resistance determinants. To this end, ECDC is undertaking pan-European molecular epidemiology surveys. In 2012, addressing the most threatening carbapenem resistance problem, the ECDC commissioned the European Survey of Carbapenemase-producing *Enterobacteriaceae* (EuSCAPE) among a consortium of hospitals to assess the prevalence and geographical distribution of carbapenemase-producing *Enterobacteriaceae* (CPE) characterized at the genetic level across 38 countries (42, 43). The project demonstrated the feasibility of conducting integrated epidemiological and microbiological sentinel multi-center surveys and of collecting quality-assured data for EU level analyses (43–45). It also built national capacities across Europe for standardized laboratory detection, identification, and surveillance of CPE (42). In 2015, all EU/EEA countries had nominated a national reference laboratory and operated national surveillance for CPE (44). The EuSCAPE surveys revealed a rapid dissemination of carbapenemase-producing *Klebsiella pneumoniae* around Europe, with wide variations by country in the type of carbapenemase, and the number of countries reaching an inter-regional or endemic level doubling from 6 to 13 countries over the period 2013–2015 (44, 45).

Rising multidrug resistance also imperils the control of gonococcal infections worldwide. Since 2009, Euro-GASP monitors emerging resistance in *Neisseria gonorrhoeae* to therapeutic antimicrobials. The program is using a standardized methodology across the EU/EEA which integrates epidemiological and microbiological data to understand the risk factors associated with resistance and thereby inform intervention strategies (39). For AST, the Euro-GASP uses a hybrid approach of EU centralized and decentralized national testing (39). EQA and training activities helped standardize surveillance across Europe: between 2009 and 2014, the number of EU/EEA countries participating in Euro-GASP increased from 17 to 23 countries while the number of countries providing quality-assured (decentralized) testing increased from 3 to 17. In addition, Euro-GASP also piloted molecular typing to unravel associations between clonal type and antimicrobial resistance profiles that could aid understanding of the dissemination of resistance within at risk populations (46). Euro-GASP results have revealed rapidly changing patterns of *N. gonorrhoeae* resistance to drugs of choice across Europe, leading to the revision of European and national gonorrhea treatment guidelines and development by ECDC of the “Response plan to control and manage the threat of multidrug-resistant gonorrhoea in Europe” (47).

## Integration of Molecular and Genomic Typing in European Surveillance and Epidemic Investigations

Whole-genome sequencing is empowering high-precision infectious disease surveillance and control (48). ECDC developed multi-annual roadmaps for the integration of molecular typing data on high-priority pathogens into its EU surveillance and epidemic preparedness systems, fact-checking the feasibility, and capacity in the EU countries, in synergy with third-party activities along a global One-Health partnership (49–51). The added-value of molecular surveillance and WGS-based outbreak investigation was demonstrated following the introduction among the ECDC FWD public health laboratory surveillance network of a novel Multi-Locus Variable Number Tandem Repeat Analysis (MLVA) typing scheme for *Salmonella* Enteritidis (52). Increases in salmonellosis cases with an uncommon MLVA profile were posted first by Scotland and then by the Netherlands in January and August 2016 in the Epidemic Intelligence Information System (53, 54). Several other countries reported detection of this MLVA profile. WGS analysis performed either by national reference laboratories or in a central facility through ECDC support confirmed a multi-country outbreak involving two distinct *Salmonella* Enteritidis genomic types. An outbreak investigation team involving affected countries, ECDC, and EFSA agreed on the outbreak case definition based on WGS and MLVA types, assessed the magnitude of the outbreak, and identified response options. At least 18 EU/EEA countries were affected by the outbreak. Environmental and food investigations by the food safety authorities showed that food establishments or retail shops in eight EU countries had received eggs contaminated with *Salmonella* Enteritidis of the epidemic MLVA or WGS types from farms located in one EU country (53).

## Outlook

Whether for health protection or for food safety, we rely on microbiology laboratories to detect, identify, and characterize human pathogens of public health significance. Although health services are a national responsibility in the EU, a cost–benefit analysis study by the European Commission has concluded that the benefits of maintaining EU public health reference laboratory networks were likely to outweigh the costs, both from a Member State and from an EU perspective (55). As outlined in the examples above, EU laboratory networks coordinated by ECDC as well as the European Commission over the last years have brought public health added value through fit-for-purpose harmonization and validation of laboratory methods, quality assurance and training activities as well as contribution to preparedness and response for biological threats. The EULabCap surveys concluded that the EU/EEA as a whole has a strong and improving public health microbiology system (7). Its key assets include harmonized methods for AST, extensive reference laboratory services, and laboratory inputs within national and EU surveillance networks. The use of molecular typing for surveillance and laboratory participation in outbreak response showed significant progress. In an era of rapid progress in electronic recording and transmission of health data, it is noticeable that half of the EU/EEA countries have implemented automated electronic reporting of microbiology laboratory data to their national communicable disease or antimicrobial resistance surveillance systems (7). To track the transmission of resistance genes needed for targeted control measures, ECDC has developed a protocol for genomic-based surveillance of carbapenem-resistant and/or colistin-resistant *Enterobacteriaceae* at the EU level (56). To deliver their full potential, these encouraging developments require sustained innovation in molecular diagnostic and typing methods and the collaborative engineering of wider connectivity between laboratory and public health information systems from the local to the global level. Collaboration between clinical and public health practitioners, government agencies, and academic institutions is key to develop integrated clinical, epidemiological, and molecular microbiological data collection, recording and exchange protocols as well as build the integrated information technology infrastructure enabling real time, molecular epidemiologic surveillance, and alert systems for infectious diseases. ECDC as convener, funding source and coordinator in close partnership with multidisciplinary experts from EU disease networks continues to act as catalyst for translating these novel approaches into practice and further strengthening the collective laboratory capability for disease control in the EU.

## Author Contributions

BA: data collation, analysis, and writing the initial draft. JR and KL: data collation and reviewing the initial draft. MS: conceptualization and rewriting the final draft.

## Conflict of Interest Statement

The authors are employees of the European Centre for Disease Prevention and Control. They declare no commercial or financial conflict of interest.
